# Supplementary material for: Analysis of PPARγ Signaling Activity in Psoriasis
Source: Int J Mol Sci. 2021 Aug 10;22(16):8603. doi: 10.3390/ijms22168603 (PMC8395241; doi:10.3390/ijms22168603)
Supplement: Supplementary file 1 [file ijms-22-08603-s001.zip › Supplemental materials_Analysis of PPARg signaling activity in psoriasis/Pathway models/Models images and html files/Model of PPARG related pathways in psoriasis (short version)/43673.html]

RPTOR


# Protein RPTOR

|  |  |
| --- | --- |
| URN | urn:agi-llid:57521 |
| Total Entities | 0 |
| Connectivity | 1344 |
| Name | RPTOR |
| Description | regulatory associated protein of MTOR complex 1 |
| Notes | This gene encodes a component of a signaling pathway that regulates cell growth in response to nutrient and insulin levels. The encoded protein forms a stoichiometric complex with the mTOR kinase, and also associates with eukaryotic initiation factor 4E-binding protein-1 and ribosomal protein S6 kinase. The protein positively regulates the downstream effector ribosomal protein S6 kinase, and negatively regulates the mTOR kinase. Multiple transcript variants encoding different isoforms have been found for this gene. [provided by RefSeq, Sep 2009] |
| Primary Cell Localization | Cytoplasm |
| ObjectType | Protein |

---

|  |  |
| --- | --- |
| Pathway | Oncogene Induced Cellular Senescence |
|  | DNA Persistent Repair Inhibits mTOR Signaling |
|  | mTOR Signaling Activation by Fatty Acids and Glucose |
|  | mTOR Signaling Activation by Amino Acids |
|  | mTOR Signaling |
|  | mTOR Signaling Hyperfunction |
|  | Hypoxia Inhibits mTOR Signaling |
|  | mTOR Signaling Activation by Taste Sensor Receptors (Rodent Model) |
|  | MTOR/TP53 Regulates Cell Metabolism |
|  | Insulin Influence on Protein Synthesis |
|  | SCF/BTRC Complex |
|  | Preconditioning Ischemia |
|  | Peutz-Jeghers Syndrome |
|  | Polycystins Mutation Effects in Cystic Kidney Disease |
|  | NOTCH1 -> Expression Targets in Colon |
|  | MTOR/TORC in Cancer Cell Motility and Invasion |
|  | Respiratory Chain Impairment in Cancer |
|  | AMPK Signaling |
|  | mTOR Signaling |
|  | ace2 |
|  | Model of PPARG signaling in psoriasis |
|  | Model of PPARG related pathways in psoriasis (short version) |

---

|  |  |
| --- | --- |
| MedScan ID | 57521 |

---

|  |  |
| --- | --- |
| LocusLink ID | 57521 |
|  | 74370 |
|  | 287871 |

---

|  |  |
| --- | --- |
| Alias | RPTOR |
|  | KOG1 |
|  | raptor |
|  | regulatory associated protein of MTOR, complex 1 |
|  | LOC653386 |
|  | LOC654218 |
|  | KIAA1303 |
|  | mKIAA1303 |
|  | OTTMUSP00000004362 |
|  | 4932417H02 |
|  | regulatory associated protein of mtor |
|  | p150 target of rapamycin (TOR)-scaffold protein |
|  | RGD1311784 |
|  | p150 target of rapamycin (TOR)-scaffold protein containing WD-repeats |
|  | 4932417H02Rik |
|  | regulatory associated protein of mammalian target of rapamycin |
|  | RP24-225K18.1 |
|  | Mip1 |
|  | regulatory-associated protein of mTOR |
|  | Rap |

---

|  |  |
| --- | --- |
| GO ID | 0071889 |
|  | 0001030 |
|  | 0001031 |
|  | 0001032 |
|  | 0001156 |
|  | 0030674 |
|  | 0030295 |
|  | 0019901 |
|  | 0030291 |
|  | 0044877 |
|  | 0031929 |
|  | 0038202 |
|  | 0032147 |
|  | 0007050 |
|  | 0071230 |
|  | 0071233 |
|  | 0031669 |
|  | 0009267 |
|  | 0071901 |
|  | 1900087 |
|  | 0032008 |
|  | 0030307 |
|  | 0001938 |
|  | 0033138 |
|  | 0010800 |
|  | 0071902 |
|  | 0045945 |
|  | 0001558 |
|  | 0008361 |
|  | 1900034 |
|  | 0016241 |
|  | 0016032 |
|  | 0031931 |
|  | 0005737 |
|  | 0010494 |
|  | 0005829 |
|  | 0030425 |
|  | 0005765 |
|  | 0005764 |
|  | 0043025 |
|  | 0005654 |
|  | 0046676 |
|  | 0042325 |
|  | 0001932 |
|  | 0032403 |
|  | 0016049 |
|  | 0016236 |
|  | 0043231 |
|  | 0034605 |
|  | 0008286 |

---

|  |  |
| --- | --- |
| KEGG ID | hsa:57521 |

---

|  |  |
| --- | --- |
| Organism | Homo sapiens {Organism urn:agi-taxid:9606} |
|  | Mus musculus {Organism urn:agi-taxid:10090} |
|  | Rattus norvegicus {Organism urn:agi-taxid:10116} |
|  | Homo sapiens |
|  | Mus musculus |
|  | Rattus norvegicus |

---

|  |  |
| --- | --- |
| Mouse chromosome position | 11 |

---

|  |  |
| --- | --- |
| OMIM ID | 607130 |

---

|  |  |
| --- | --- |
| Rat chromosome position | 10q32.3 |

---

|  |  |
| --- | --- |
| Hugo ID | 30287 |
|  | HGNC:30287 |

---

|  |  |
| --- | --- |
| Human chromosome position | 17q25.3 |

---

|  |  |
| --- | --- |
| Swiss-Prot Accession | Q8N122 |
|  | Q6DKI0 |
|  | Q8N122.1 |
|  | A2ACM0 |
|  | A4FUW1 |
|  | Q8K4Q0.1 |
|  | D3ZDU2 |
|  | B2RN36 |
|  | C6KEF2 |
|  | F5H7J5 |
|  | Q8N4V9 |
|  | Q8TB32 |
|  | Q9P2P3 |
|  | Q8K4Q0 |

---

|  |  |
| --- | --- |
| GenBank ID | NC\_000017 |
|  | NM\_020761 |
|  | NP\_065812 |
|  | NM\_001163034 |
|  | NP\_001156506 |
|  | NG\_013034 |
|  | AC016245 |
|  | AC109327 |
|  | AC127496 |
|  | AC133012 |
|  | CH471099 |
|  | EAW89618 |
|  | EAW89619 |
|  | KC877680 |
|  | AB037724 |
|  | BAA92541 |
|  | AB082951 |
|  | BAC06490 |
|  | AI821182 |
|  | AK055912 |
|  | AK295964 |
|  | BAG58742 |
|  | AK296321 |
|  | BAH12314 |
|  | AY090663 |
|  | AAM09075 |
|  | BC014502 |
|  | BC025180 |
|  | AAH25180 |
|  | BC033258 |
|  | AAH33258 |
|  | BC041079 |
|  | BC064515 |
|  | AAH64515 |
|  | BC073898 |
|  | AAH73898 |
|  | BC136652 |
|  | AAI36653 |
|  | BC136654 |
|  | AAI36655 |
|  | BF111734 |
|  | GQ183898 |
|  | ACS44766 |
|  | Q8N122 |
|  | NC\_000077 |
|  | NM\_028898 |
|  | NP\_083174 |
|  | NM\_001306081 |
|  | NP\_001293010 |
|  | XM\_006534363 |
|  | XP\_006534426 |
|  | XM\_006534361 |
|  | XP\_006534424 |
|  | XM\_006534362 |
|  | XP\_006534425 |
|  | XM\_011249286 |
|  | XP\_011247588 |
|  | XM\_011249285 |
|  | XP\_011247587 |
|  | AL669881 |
|  | AL928989 |
|  | AL929234 |
|  | BX248132 |
|  | CH466558 |
|  | EDL34713 |
|  | EDL34714 |
|  | EDL34715 |
|  | AB082952 |
|  | BAC06491 |
|  | AK016530 |
|  | BAB30288 |
|  | AK029341 |
|  | AK034306 |
|  | BAC28669 |
|  | AK034557 |
|  | BAC28753 |
|  | AK034733 |
|  | AK040288 |
|  | BAC30561 |
|  | AK046898 |
|  | AK081250 |
|  | AK087217 |
|  | AK129326 |
|  | BAC98136 |
|  | AK137878 |
|  | AK144082 |
|  | AK161011 |
|  | AK172276 |
|  | BC023733 |
|  | BC052202 |
|  | BC059877 |
|  | BC060140 |
|  | BC118971 |
|  | AAI18972 |
|  | BC148309 |
|  | AAI48310 |
|  | Q8K4Q0 |
|  | NC\_005109 |
|  | NM\_001134499 |
|  | NP\_001127971 |
|  | XM\_017597164 |
|  | XP\_017452653 |
|  | XM\_017597165 |
|  | XP\_017452654 |
|  | AC\_000078 |
|  | AABR07030912 |
|  | AABR07030913 |
|  | AABR07030914 |
|  | AAHX01066964 |
|  | AAHX01066965 |
|  | AAHX01066966 |
|  | AAHX01066967 |
|  | AAHX01066968 |
|  | AAHX01066969 |
|  | AAHX01066970 |
|  | AAHX01066971 |
|  | AAHX01066972 |
|  | CH473948 |
|  | EDM06799 |
|  | NC\_018928 |
|  | AMYH02034109 |
|  | AMYH02034110 |
|  | AMYH02034111 |
|  | AMYH02034112 |
|  | AMYH02034113 |
|  | AMYH02034114 |
|  | AMYH02034115 |
|  | AMYH02034116 |
|  | AMYH02034117 |
|  | BC033699 |
|  | XM\_006534364 |
|  | XP\_006534427 |
|  | AC\_000033 |
|  | AAHY01096916 |
|  | AAHY01096917 |
|  | AAHY01096918 |
|  | AAHY01096919 |
|  | AAHY01096920 |
|  | AAHY01096921 |
|  | AAHY01096922 |
|  | AAHY01096923 |
|  | AAHY01096924 |
|  | AAHY01096925 |
|  | AAHY01096926 |
|  | BC167200 |
|  | AAI67200 |
|  | AC\_000149 |
|  | ABBA01032115 |
|  | ABBA01032116 |
|  | ABBA01032117 |
|  | ABBA01032118 |
|  | ABBA01032119 |
|  | ABBA01032120 |
|  | ABBA01032121 |
|  | ABBA01032122 |

---

|  |  |
| --- | --- |
| Swiss-Prot ID | RPTOR\_HUMAN |

---

|  |  |
| --- | --- |
| Cell Localization | Cytoplasm |
|  | Lysosome |
|  | Cytoplasmic granule |

---

|  |  |
| --- | --- |
| Ensembl ID | ENSG00000141564 |
|  | ENSP00000442479.2 |
|  | ENST00000544334.6 |
|  | ENSP00000307272.3 |
|  | ENST00000306801.8 |
|  | ENSMUSG00000025583 |
|  | ENSMUSP00000026671.6 |
|  | ENSMUST00000026671.12 |
|  | ENSRNOG00000003821 |
|  | ENSRNOP00000005337.5 |
|  | ENSRNOT00000005337.6 |
|  | ENSP00000442479 |
|  | ENST00000544334 |
|  | ENSP00000307272 |
|  | ENST00000306801 |
|  | ENSMUSP00000026671 |
|  | ENSMUST00000026671 |
|  | ENSRNOP00000005337 |
|  | ENSRNOT00000005337 |

---

|  |  |
| --- | --- |
| MGI ID | MGI:1921620 |

---

|  |  |
| --- | --- |
| RGD ID | 1311784 |

---

|  |  |
| --- | --- |
| Unigene ID | Hs.133044 |
|  | Mm.209933 |
|  | Mm.442144 |
|  | Mm.450470 |
|  | Mm.462932 |
|  | Rn.98539 |

---

|  |  |
| --- | --- |
| Homologene ID | 80210 |

---
